# Ampelopsin targets in cellular processes of cancer: Recent trends and advances

**DOI:** 10.1016/j.toxrep.2022.07.013

**Published:** 2022-07-27

**Authors:** Hardeep Singh Tuli, Katrin Sak, Vivek Kumar Garg, Ajay Kumar, Shubham Adhikary, Ginpreet Kaur, Nidarshana Chaturvedi Parashar, Gaurav Parashar, Tapan Kumar Mukherjee, Uttam Sharma, Aklank Jain, Ranjan K. Mohapatra, Kuldeep Dhama, Manoj Kumar, Tejveer Singh

**Affiliations:** a Department of Biotechnology, Maharishi Markandeshwar (Deemed to be University), Mullana-Ambala, Haryana 133207, India; b NGO Praeventio, Tartu 50407, Estonia; c Department of Medical Laboratory Technology, University Institute of Applied Health Sciences, Chandigarh University, Gharuan, Mohali 140413, Punjab, India; d Department of Botanical and Environmental Sciences, Guru Nanak Dev University, Amritsar 143005, Punjab, India; e Shobhaben Pratapbhai Patel School of Pharmacy and Technology Management, SVKM’s NMIMS, Mumbai 40056, Maharashtra, India; f Department of Zoology, Central University of Punjab, Village-Ghudda, 151401 Punjab, India; g Department of Chemistry, Government College of Engineering, Keonjhar 758002, Odisha, India; h Division of Pathology, ICAR-Indian Veterinary Research Institute, Bareilly, Uttar Pradesh 243122, India; i Department of Chemistry, Maharishi Markandeshwar University, Sadopur-Ambala 134007, Haryana, India; j School of life Science, Jawaharlal Nehru University, New Delhi, India

**Keywords:** Ampelopsin, Anti-proliferation, Apoptotic, Cell cycle arrest, Angiogenesis inhibition, Anti-inflammation

## Abstract

Cancer is being considered as a serious threat to human health globally due to limited availability and efficacy of therapeutics. In addition, existing chemotherapeutic drugs possess a diverse range of toxic side effects. Therefore, more research is welcomed to investigate the chemo-preventive action of plant-based metabolites. Ampelopsin (dihydromyricetin) is one among the biologically active plant-based chemicals with promising anti-cancer actions. It modulates the expression of various cellular molecules that are involved in cancer progressions. For instance, ampelopsin enhances the expression of apoptosis inducing proteins. It regulates the expression of angiogenic and metastatic proteins to inhibit tumor growth. Expression of inflammatory markers has also been found to be suppressed by ampelopsin in cancer cells. The present review article describes various anti-tumor cellular targets of ampelopsin at a single podium which will help the researchers to understand mechanistic insight of this phytochemical.

## Graphical Abstract

**Figure:**
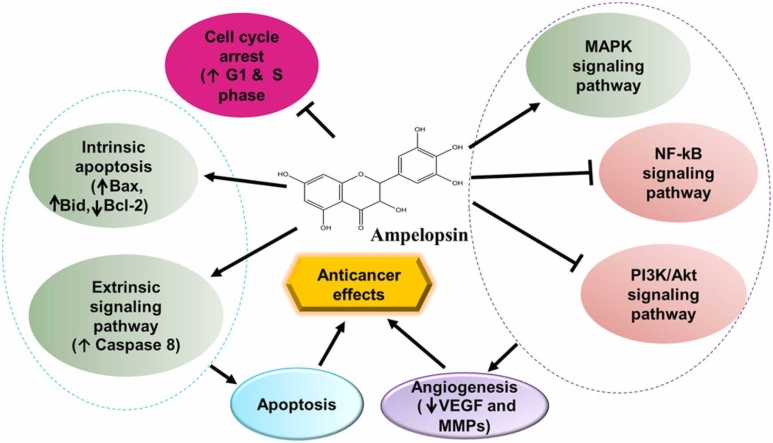

## 1 Introduction

The incidence of cancer diseases is continually increasing worldwide to cause millions of deaths each year. Based on the latest estimates, the global cancer burden will considerably increase over the next decades, being expected to reveal a 47% rise by 2040 as compared to the year 2020 [1], [2], [3], [4]. Due to various shortcomings in the traditional therapeutic formulations, more and more researchers all over the world are working on identifying novel anticancer agents and developing new efficient modalities to manage this dreadful disease [5], [6], [7]. A great majority of the current clinically approved chemotherapeutic drugs was came from various natural sources, from microbes, terrestrial and marine plants [8], [9], [10], [11]. In fact, well-known medications such as vinblastine and vincristine, paclitaxel and podophyllotoxin were all discovered in plants [8]. Being inspired by this initial success, many research groups around the world are devoted to separation of novel structural leads from different plant species and testing them regarding potential anticancer activities [12], [13], [14], [15].

One of such promising compounds is ampelopsin, also termed dihydromyricetin, that was first isolated from the plant *Ampelopsis meliaefolia* Kudo already in 1940, and was later detected as a major bioactive ingredient in *Ampelopsis grossedentata* (Handel-Mazzetti) W. T. Wang [16]. Several preclinical studies have displayed the ability of ampelopsin to suppress the growth of breast [17], ovarian [18], prostate [19], lung [20], colon [21], liver [22], renal [23] and bladder cancer [24] as well as glioma [25], osteosarcoma [26] and leukemia cells [27] via modulating various cellular signaling pathways. Ampelopsin has been indeed shown to inhibit proliferation, induce apoptosis, suppress migration, invasion and metastasis in various types of tumoral cells [17], [18], [27], [28]. Moreover, treatment of malignant cells with ampelopsin was also demonstrated to confer overcoming resistance and potentiating the responses to standard anticancer drugs like erlotinib [29] or carboplatin [30], opening new avenues in cancer treatment. Therefore, in this review article the molecular mechanisms behind these promising effects are described in depth. In addition, modern nanotechnological opportunities are discussed for enhancing the bioavailability of ampelopsin and improving its anticancer potential in vivo conditions. It is expected that intensifying the studies of natural anticancer agents will ultimately get us a step closer to solving the riddle of successful cancer therapy. Table 1, Table 2 gives a bird eye view of various in vivo and in vitro antiproliferative actions of ampelopsin.

**Table 1:** An overview of in vitro anticancer potential of Ampelopsin.

Type Of Cancer	Cell Lines	Effects	Mechanisms	References
Leukemia	HL60 and K562	Induces Apoptosis	↓AKT and NF-κB	[27]
Hepatoma	HepG2	Induces Apoptosis	↑ERK1/2 and P38	[31]
Glioma	U251 and A172	Induces Apoptosis	↑JNK, G1 and S phase arrest	[32]
Breast Cancer	MDA-MB-231 and MCF-7	Induces Apoptosis	↑ Bax/Bcl-2	[17]
Pheochromocytoma	PC12	Induces Apoptosis	↑ERK and Akt	[33]
Human Lung Adenocarcinoma	A549	Induces Apoptosis	↓ c-Myc/Skp2 and HDAC1	[34]
Cervical Cancer	HeLa	Induces Apoptosis	↑ Bax/Bcl-2	[35]
Renal Cell Carcinoma	786-O cells.	Induces Apoptosis, inhibits cell viability and metastasis	↓PI3K/AKT	[23]
Breast Cancer	MDA-MB-231	Induces Apoptosis	↓TNF-α/NF-κB	[36]
Breast Cancer	MDA-MB-231 and MCF-7	Induces Apoptosis	↓GRP78 and CHOP	[37]
colon cancer	HCT-116, HCT-8 and HT-29	Induces Apoptosis	↑AMPK/MAPK/XAF1	[21]
Breast Cancer	MDA-MB-231 and MCF-7	Induces Autophagy	↓Akt-mTOR	[38]
osteosarcoma	MG-63 cells	Induces Apoptosis	↑p21(CIP1) and G0/G1 phase arrest	[26]
non-small cell lung cancer	H1975 and H1650	Induces Apoptosis	↑Nox2-Bim	[29]
Breast Cancer	MDA-MB-231	Induces Apoptosis	↓AxL, TYRO3, and FYN	[39]
Breast Cancer	MCF-7	Induces Apoptosis (with Resveratrol, ampelopsin A and balanocarpol)	↓sphingosine kinase 1	[40]
Breast Cancer	MDA-MB-231	Induces Apoptosis	↓mTOR	[41]
Breast Cancer	MDA-MB-231	induces Apoptosis	G2/M arrest	[42]
human lung adenocarcinoma	SPC-A-1 cell	induces Apoptosis	promoted tubulin polymerization	[43]
human bladder carcinoma and murine sarcoma	EJ cells (human) and 180 cells (Murine)	Inhibited proliferation	cell cycle arrest	[24]
Prostate cancer	PC-3 and LNCaP	Inhibits migration, invasion and metastasis	↓Bcl-2	[19]
Melanoma	B16	Inhibits metastasis		[44]
ovarian cancer cell	A2780 cells	Proliferation, migration and invasion	inhibited EMT, ↓NF-κB/Snail pathway	[18]
Melanoma	B16	Inhibits, migration, invasion, and metastasis		[45]

**Table 2:** *In vivo* anticancer action of Ampelopsin.

Type of Cancer	subject model	Effects	Mechanisms	References
Glioma	U251 and A172	Induces Apoptosis	↑JNK, G1 and S phase arrest	[25]
Breast Cancer	MDA-MB-231	Induces Apoptosis	↓mTOR	[41]
human bladder carcinoma and murine sarcoma	EJ cells (human) and 180 cells (Murine)	Inhibited proliferation	cell cycle arrest	[24]
Prostate cancer	PC-3 cells	Induces apoptosis and inhibits proliferation, reduces prostate tumor angiogenesis	↓ CXCR4	[19]
Melanoma	B16	Inhibits metastasis		[44]
Melanoma	B16	Inhibits, migration, invasion, and metastasis		[45]

## 2 Chemistry of ampelopsin

**Fig. 1:**
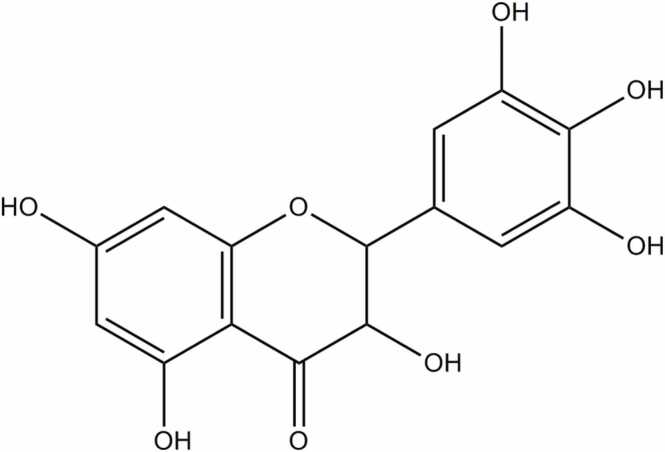
Chemical structure of Ampelopsin.

The chemical name of ampelopsin is 3, 5, 7, 3′, 4′, 5′-hexahydroxyl-2, 3-dihydroflavonol (Fig. 1), initially isolated from traditional Chinese medicinal plants *Ampelopsis grossedentata* and *Ampelopsis meliaefolia*. It is a major bioactive constituent approximately 30–40% (w/w) of *Ampelopsis grossedentata*. It has also been reported in other plants/ plants-based foods such as *Hovenia dulcis* and *Cedrus deodara*/ grapes and red bayberry [46].

**Fig. 2:**
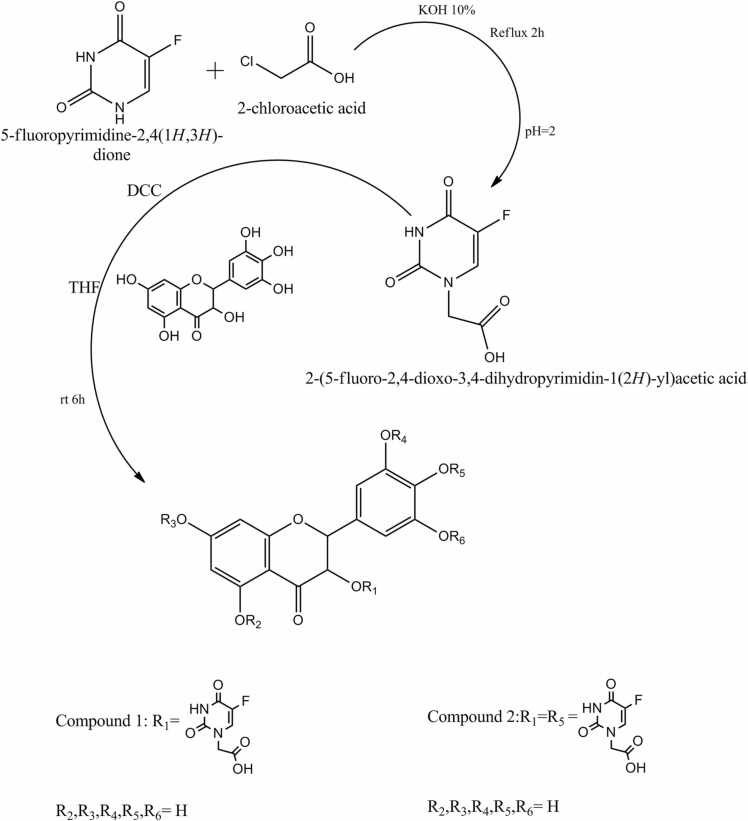
**:** Schematic representations of two novel 5-fluorouracil-substituted ampelopsin derivatives.

Zhou et al. investigated laboratory based synthetic methods of two novel 5-fluorouracil-substituted ampelopsin derivatives from 5-fluorouracil-1-carboxylic acid [47]. They established the structures of synthesized compounds by spectroscopic and elemental analysis methods (Fig. 2). Researchers found that new 5-FU-substituted ampelopsin derivatives were more effective anticancer agents against K562 and K562/ADR, cancer cell lines in comparison to native moiety.

## 3 Pharmacokinetics of ampelopsin

Pharmacokinetic investigations have indicated that the maximal plasma concentrations are 81.3 and 107 ng/ml of dextroisomer and racemate ampelopsin, respectively. This was after oral administration of 100 mg/kg [48]. The half-lives during degradation were recorded to be 288 and 367 min. This confirms weak absorption and rapid degradation nature of ampelopsin into the bloodstream [49]. According to Fan et al., unconverted ampelopsin is promptly distributed in the gastrointestinal system. It was also discovered that ampelopsin gets metabolized to three components by microflora through dihydroxylation and reduction [49].

Ampelopsin is also capable of crossing the blood-brain barrier. Xiang et al. investigated poor absorption nature of ampelopsin in Caco-2 cells, and lowering in the pH from 8.0 to 6.0 significantly improved its absorption [50]. Similarly, the pH of the solution has a significant impact on the intestinal stability of ampelopsin [51]. It was steady in a pH range of 1.0–5.0 in the stomach, whereas partial degradation occurred at a pH of 6.8 in the intestinal environment even though ampelopsin metabolites were only detectable in negligible concentrations in plasma, urine and feces revealed a total of eight metabolites [51].

Glucuronidation and sulfation metabolites have been observed in urinary samples, while dihydroxylation and reduction based intermediates were only found in feces [52]. Past research has shown that methylation pathways may be found in both urinary and fecal samples. Furthermore, some unchanged ampelopsin is also eliminated in the fecal samples [50]. Flavonoids' based intermediates are usually excreted through the biliary or urine systems [53]. The biliary system is in charge of excretion of both unchanged ampelopsin and its intermediates, while ampelopsin metabolism involves the urinary system. Significant enhancement of detection peak of ampelopsin levels in germ free rat vs. controlled models implicated the pharmacokinetic modulatory action of gut microbiota [53]. Due to its low water solubility, the bioavailability of ampelopsin is poor. Its absolute bioavailability in rats was found to be only less than 10% [54]. This shows a high need for improving the bioavailability to apply all beneficial pharmacological effects of ampelopsin in vivo conditions in the future.

## 4 Chemo-preventive action of ampelopsin

### 4.1 Induction of apoptosis and cell cycle arrest

**Fig. 3:**
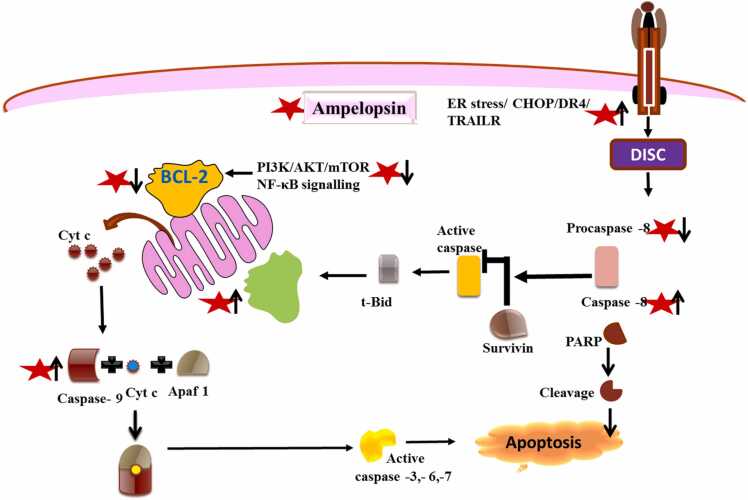
**:** Apoptotic mechanisms of action of ampelopsin in cancer. It initiates intrinsic (mitochondrial) as well extrinsic (Death receptor medicated) mechanisms of apoptosis induction.

There are multiple anti-cancer properties reported for ampelopsin, including the potential to induce apoptosis (Fig. 3). In breast cancer cells (MDA-MB-231 and MCF-7), and cervical cancer (HeLa) cell line apoptotic potential of ampelopsin is imparted through mitochondrial dysfunction, loss of MMP, accumulation of reactive oxygen species, and upregulated Bax/Bcl-2 expression [17], [35]. It was shown that along with mitochondrial damage, ampelopsin also activates caspase-9,− 3 and PARP and downregulates AKT and NF-κB expressions [27]. Ampelopsin treatment reduces the phosphorylated levels of IκBα and NF-κB p65, both independent and tumor necrosis factor (TNF)-α-stimulated thus disrupting the TNF-α/NF-κB signaling axis as reported in MDA-MB-231/IR cells [36]. In lung adenocarcinoma SPC-A-1 cells, ampelopsin treatment leads to increased Ca^2+^ levels, mitochondrial nitric oxide production and decreased total ATPase activity and ΔΨm [55]. Anti-glioma role of ampelopsin was reported in human glioma (U251 and A172) cell lines (treated at 0, 25, 50, and 100 μM for 24 h) and human glioma xenograft models (50 and 100 mg/kg). Ampelopsin exerted its anti-glioma effects both through intrinsic and extrinsic pathways and upregulated c-Jun N-terminal protein kinase (JNK) expression [32]. In hepatoma HepG2 cells ampelopsin induces apoptosis through activating death receptor 4 and 5 mediated increase of Bax/Bcl-2 ratio. Moreover, minimal expression of JNK1/2 and activated p38, upon ampelopsin treatment were reported in HepG2 cells [31]. Similar report on ampelopsin induced apoptosis through upregulated TRAIL/DR5 and downstream activation of p38 signaling has been reported in Epstein-Barr virus (EBV)-positive cells [56]. In renal cell carcinoma, ampelopsin treatment at 100 μM has been reported to induce apoptosis by negatively regulating the PI3K/AKT signaling pathway [57]. In lung cancer A549 cell line, ampelopsin has been reported to induce apoptotic cell death via modulating multiple c-Myc/S-phase kinase-associated protein 2/F-box and WD repeat-containing protein 7/histone deacetylase 2 signaling mechanisms [34]. Ampelopsin has also been reported to exert its apoptosis inducing potential through endoplasmic reticulum (ER) stress pathway. In, breast cancer (MDA-MB-231 and MCF-7) cell lines, ampelopsin treatment induces ER-stress as evidenced by activated ER-stress related proteins such as including GRP78, p-PERK, p-elF2α, cleaved ATF6α and CHOP [37]. Ampelopsin induced ER-stress activates AMPK, and MAPKs pathway in colon cancer cells. RNAi mediated blocking of JNK or p-38/MAPK and not AMPK inhibits apoptosis suggesting ER-stress may be independent of AMPK induction and involve ROS mediated activation [58]. Therefore ROS production results in induction of ER stress followed by activation of MAPK signaling and apoptotic cell death via upregulation of XAF-1. Ampelopsin also showed anti-cancer potential against breast cancer (MDA-MB 231) cells via inhibition of increased mTOR activity. Possible explanation provided suggests inhibition of Akt, mTOR complexes 1/2 and decreased activation of p70-S6 kinase [41].

Ampelopsin may impart its anti-cancer activities independent of apoptosis, such as by regulating cell cycle mechanism. Ampelopsin has been reported to arrest HL60 cells at the sub-G1 phase and K562 cells in S-phase. Differential regulation may be explained on the basis of ampelopsin regulation of cyclins and CDK’s [59]. Moreover, ampelopsin-treated U251 and A172 glioma cells were observed to be arrested in G1 and S phase initiated through ROS generation and autophagy [32]. It has also been suggested that ampelopsin may induce tubulin polymerization in lung adeno carcinoma SPC-A-1 cell line which may subsequently disrupt mitosis and arrest cells in S- phase both in SPC-A-1 and Hela cells [35], [43].

### 4.2 Anti-angiogenic and anti-metastatic mechanisms

**Fig. 4:**
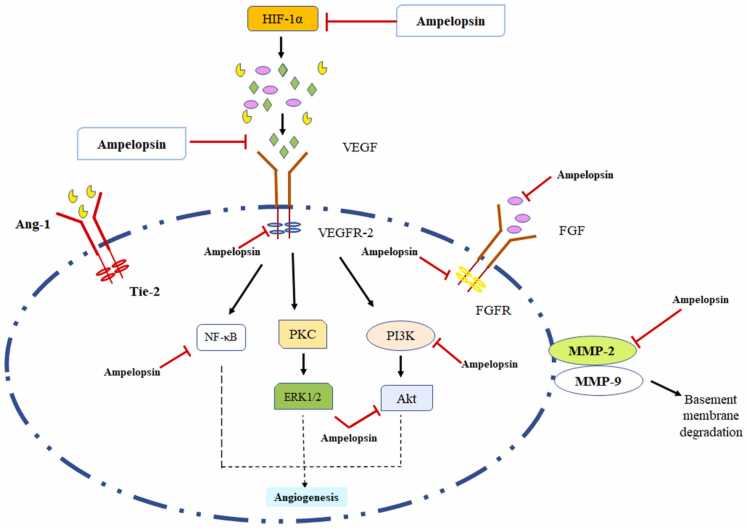
**:** Anti-angiogenic and anti-metastatic action of ampelopsin via modulation of VEGF and MMPs expression.

Neo-vasculature is the development of new blood vessels from the preexistent vessels. All living cells including tumors need oxygen and nutrients to survive and proliferate. Angiogenesis is a critical step in proliferation and metastasis of cancer cells. One indirect way to prevent cancer is through halting this process and depriving the tumor of nutrients. In addition angiogenesis is important in wound healing, reproduction and pregnancy too [60], [61], [62], [63]. Deregulated angiogenesis causes a variety of pathological conditions such as malignancies, neurodegeneration, psoriasis and proliferative retinopathy [64], [65], [66]. Tumorigenesis relies on angiogenesis because all metabolic processes such as necessary nutrients, growth hormones, and molecular oxygen are circulated by angiogenesis process [67], [68], [69]. As a result, inhibiting angiogenesis could be a viable technique for treating cancer and other disorders linked to angiogenesis in humans[15], [70]. The primary hallmarks of angiogenesis are endothelial cell proliferation, metastasis and tubulogenesis by growth factors [71], [72], [73]. [74]. VEGFR2 plays a main role in signaling that promotes the migration and proliferation of endothelial cells by activating several downstream mediators, including signal STAT 3, ERK 1/2 and AKT (Fig. 4). Matrix metalloproteinases (MMPs) are extracellular basement membrane digesting enzymes that facilitates tumor invasion and migration from one site to other [75], [76], [77]. MMP-2 and MMP-9 are known to hold promising actions in the breakdown of laminin, type IV collagen and gelatin as well as other ECM and basement membrane components [78]. Therefore, MMPs are the key players in the deprivation of basement membrane and VEGFR2-mediated cascades that are used in cancer metastasis. Hence, inhibiting VEGFR2 and MMPs signal transduction may be used as an anti-angiogenesis therapy. Naturally isolated compounds such as paclitaxel, camptothecin, combretastatin and farnesiferol C showed angiogenesis regulating properties [79], [80], [81]. Ampelopsin showed antiproliferation potential against human hepatocellular carcinoma (Bel-7402) cell through downregulation of vascular endothelial growth factor (VEGF) and Basic fibroblast growth factor (bFGF) [82]. Ni et al., 2012 reported that ampelopsin dose-dependently reduced the metastasis of prostate cancer (PC-3) cells via downregulation CXCR4 expression [19]. Zhang et al., 2014 reported that ampelopsin dose-dependently inhibits the migration of hepatoma cell lines (SK-Hep-1 and MHCC97L) via downregulation in the expression of MMP-9, p38, ERK1/2 and JNK [83]. Ampelopsin significantly upregulated E-cadherin and downregulated EMT via the NF-κB/Snail pathway in human ovarian cancer (A2780) cell line [18]. Han et al., 2017, reported that ampelopsin isolated from *Hovenia dulcis* reduced angiogenesis with no sign of toxicity in HUVECs via downregulation of VEGFR2 and hypoxia-inducible factor (HIF-1α) expression [60]. Huang et al., 2019 investigated that ampelopsin A and ampelopsin C isolated from *Vitis thunbergii* showed anti-metastatic and apoptosis-inducing against breast cancer (MDA-MB-231) cells via decreasing phosphorylation of AXL, Dtk (TYRO3), EphA2, EphA6, Fyn, Hck and SRMS [39]. Ampelopsin significantly inhibit PI3K/Akt signaling by inactivation of NF-κB and thereby reducing the MMP-9 expression leading to anti-metastasis of Leukemia Cells (HL60 and K562) [59].

### 4.3 Anti-inflammatory effect

**Fig. 5:**
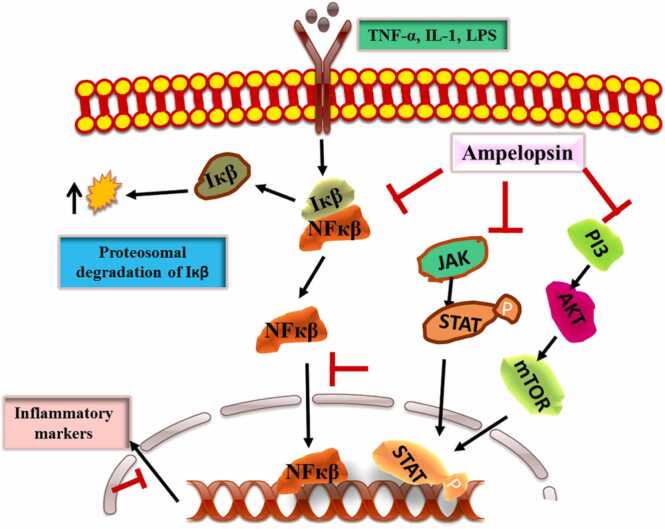
**:** Anti-inflammatory mechanisms governed by ampelopsin. It mainly regulates PI3K/Akt/NF-κB and STATs signaling to inhibit inflammation in tumor microenvironment.

Inflammation is a natural reaction of the body’s immunological cells and mediators such as cytokines (e.g. TNFα), chemokines (e.g. CCL2), reactive oxygen species (ROS) (e.g. O^−^ 2), reactive nitrogen species (RNS) (e.g. NO_3_^−^**)** etc. released by these cells to protect against injury and infections by pathologic agents such as viruses, bacteria, fungi, protozoa etc. [84], [85]. However, uncontrolled or overproduction of inflammatory mediators leads to complication and propagation of various disease conditions including different cancers [86], [87], [88], [89], [90]. The anticancer activities of ampelopsin are widely supported by its anti-inflammatory (Fig. 5) and anti-oxidative activities. The supporting evidence originated from a number of in vitro studies by using cancer cell lines. In one such study Han et al. used ampelopsin to show decreased level of cell proliferation as well as increased level of apoptosis of HL60 and KL562 leukemia cell lines by down regulating AKT and NF-kB dependent signaling pathways respectively [59]. In another study Liu et al. showed that ampelopsin attenuated TNFα-induced migration and invasion of osteosarcoma cells. The mechanism relates to the ampelopsin dependent suppression of p38MAPK/MMP-2 signaling pathways [91]***.*** In microglial cells, ampelopsin inhibited lipopolysaccharide (LPS)-induced inflammatory signals via downregulation of NF-κB and JAK2/STAT3 signaling pathways [92]***.*** Of note, NF-κB and STATs are recognized as proinflammatory transcription factors [93], [94], [95], [96]. Various levels of cross-talk exist between proinflammatory and prooxidative molecules [97]. Significantly, ampelopsin is now well accepted as a potential redox balance mediator by inhibiting LPS induced ROS generation in cancer cells [98]***.*** Studies by Qi et al. also confirmed that ampelopsin reduces endotoxic inflammation via repressing ROS-mediated activation of PI3K/Akt/NF-κB signaling pathways [99]***.*** Confirmatory in vivo studies are required to ascertain the anti-inflammatory activities of ampelopsin as one of the important mediators to attenuate various types of cancer complications.

### 4.4 Ampelopsin mediated regulation of microRNAs

Accumulating evidence revealed that human biological system contains ~98% non-coding genes and ~2% coding genes [100], [101], [102]. Due to the abundance of non-coding genes, scientists are trying to investigate the biological, molecular, and cellular roles in human physiology. Several studies have shown that microRNAs (miRNAs), a highly conserved member of non-coding genes, are 20–23 nucleotides in size and are closely associated with cancer development [103], [104]. miRNAs play a significant role in apoptosis, cell cycle regulation, cell proliferation, and metastasis of various cancer cells and thus facilitate cancer pathogenesis such as esophageal cancer [105], cholangiocarcinoma. (CCA) [106], [107]. Due to their widespread role in regulating cancer phenotypes, miRNAs can be utilized in cancer therapy. However, the literature suggests that miRNA’s efficacy can be enhanced by incorporating bioactive compound molecules [106], [107].

For example, Lei Chen et al. [107] showed anti-tumor effects of ampelopsin in two human CCA cell lines, HCCC9810 and TFK-1. The authors showed that the half-maximal inhibitory concentration (IC50) value of 150 μM of ampelopsin inhibited the HCCC9810 and TFK-1 cells proliferation by 60%, cell migration by 60% and invasion by 80% compared to control CCA cells [107]. In contrast, the apoptosis rate was increased four times compared to control CCA cells. At molecular levels, treated HCCC9810 and TFK-1 with 150 μM of ampelopsin significantly enhanced the protein levels of cleaved caspase-3 and Bad as well as inhibited the protein levels of Bcl-2 and matrix metalloproteinase (MMP9) and Vimentin compared to the endogenous level in the control cells [107], suggesting the role of ampelopsin in cell proliferation, apoptosis, migration and invasion in CCA. Mechanistically, ampelopsin possesses anti-cancer effects in CCA through negatively regulating the well-known miR-21. miR-21 acts as an oncogene in CCA, and their oncogenic property gets abrogated by incorporating ampelopsin in a dose-dependent manner through the miR-21/phosphatase and tensin homolog deleted on chromosome 10 (PTEN)/protein kinase B (Akt) pathway [107]. Interestingly, HCCC9810 and TFK-1 cells treated with ampelopsin promoted the protein level of PTEN and inhibited the phosphorylated Akt (p-Akt). However, reverse effects were demonstrated in the protein level of PTEN and p-Akt in cells with overexpressed miR-21 levels [107].

Moreover, studies showed that ampelopsin not only acts through oncogenic miRNA but also through negatively targeting tumor suppressor miR-455–3p in CCA cells [106]. Interestingly, miR-455–3p executes its function by negatively regulating zinc finger E-box-binding homeobox 1 (ZEB1) expression in CCA cells RBE and TFK-1. Furthermore, inhibition of miR-455–3p expression abolished the tumor-suppressing property of ampelopsin on cell proliferation, migration, and invasion in RBE and TFK-1 cells via PI3K/Akt mechanisms [106]. Mechanistically, the inhibitory effect of ampelopsin was abrogated by the downregulation of miR-455–3p and hence increased the protein expression levels of mesenchymal marker ZEB1 and Vimentin and suppressed the protein expression levels of mesenchymal marker E-Cadherin [106].

**Fig. 6:**
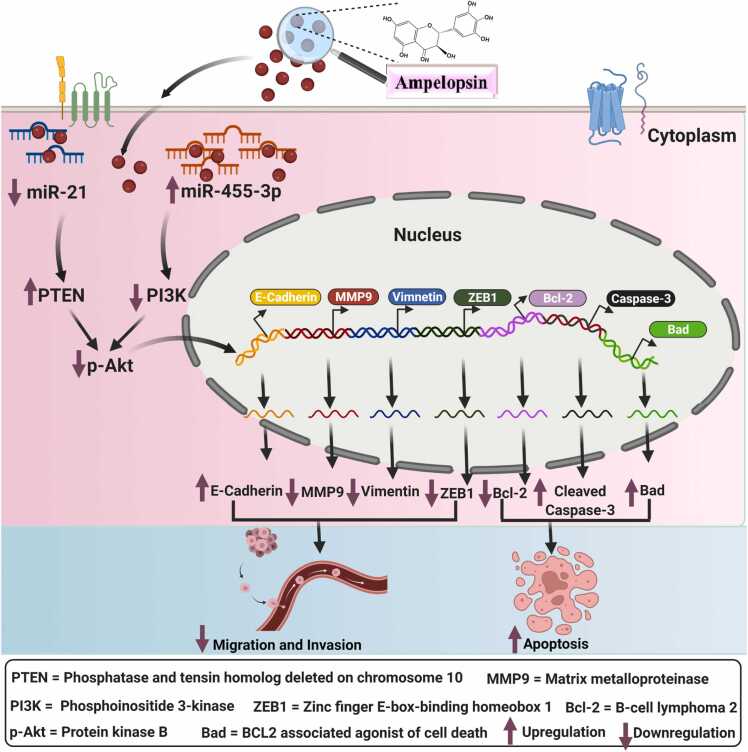
**:** Ampelopsin incorporation into the cancer cells attenuates the oncogenic effect of miR-21 and tumor-suppressive effect of miR-455–3p through PTEN and PI3K pathway, which results in the activation of protein kinase B (p-Akt), which leads to the sequestration of mesenchymal markers and anti-apoptotic genes and enrichment of epithelial markers and pro-apoptotic genes.

Overall, ampelopsin exerts great anti-tumorigenic effects on cell proliferation and metastasis through regulating the miR-21/PTEN/Akt pathway and miR-455–3p/ZEB1/PI3K/Akt pathway (Fig. 6) in CCA, which provides an alternative option for the future treatment of cancer.

## 5 Synergistic applications of ampelopsin

Phytochemicals such as flavonoids are known to possess anti-tumor effect with lower side effects in comparison to chemotherapeutics [14], [108]. Various synergistic chemo-preventive techniques with greater sensitivity in combination with known chemotherapeutic medications have attracted a lot of interest in recent years [109], [110], [111], [112]. Ampelopsin has been well demonstrated to exhibit potent cancer chemopreventive efficiency through regulation of different mechanisms [36] and also manifest hypoglycemic, antioxidant, antiviral and hepato-protective activities [35]. Furthermore, research suggests that ampelopsin improves the efficacy of some medications that could be used in chemoprevention and anticancer therapy. The drug was demonstrated to have a synergistic effect with irinotecan (CPT-11) or gemcitabine (GM), in an AOM/DSS-induced colitis-associated colon cancer model and a Min (Apc Min/+) mouse model, suggesting that a sufficient dose of ampelopsin could operate as a co-adjuvant to CPT-11 chemotherapy [113]. Furthermore, studies have also revealed that a combination of ampelopsin with ERK, JNK inhibitors and the reactive oxygen species scavenger, NAC resulted in reversal of ampelopsin-induced ERK and JNK activation and also sensitized the anti-tumorigenic effect of ampelopsin on Non-small cell lung cancer (NSCLC) cells, thus prove to be an effective strategy for preventing NSCLC proliferation [114]. Due to possibility of tumors acquiring chemotherapeutic drug resistance cancer management sometimes becomes more challenging.

As a result, combination therapeutic techniques have emerged as a preferable option because they not only minimize the amount of chemotherapy necessary, i.e. for improving chemo-sensitivity, but they also amplify the anticancer effect of regular medications [109], [115], [116], [117]. Likewise, combinational effects of ampelopsin with oxaliplatin (OXA) have been found to increase OXA-induced apoptosis and reduced 5(6)-carboxy-2′,7′-dichlorofluorescein accumulation in OXA-resistant colorectal cancer (CRC HCT116/L-OHP) cells, thus indicating inhibition of multidrug resistance protein 2 (MRP2) mediated MDR, enhancement of chemo sensitivity and increasing anticancer activity induced by oxaliplatin in colorectal cancer cells [118]. Moreover, ampelopsin has also been found to potentiate retinoic acid mediated differentiation therapy for leukemia patients. It was observed that ampelopsin sensitized the APL (NB4) cells to ATRA-induced cell growth inhibition, CD11b expression, NBT reduction and myeloid regulator expression, thus posing possibility to explore ampelopsin and retinoic acid therapy [119]. Additionally, this natural flavonoid compound, in synergism with an improved platinum-containing drug, In hepatocellular carcinoma (HCC) patients, nedaplatin (NDP) has also been demonstrated to improve tumor susceptibility to chemotherapeutics and to prevent significant hepatocyte damage as well as drug resistance. The combination of ampelopsin and nedaplatin was shown to activate the p53/Bcl-2 signaling pathway, resulting in mitochondrial malfunction, cell death, and growth inhibition in HCC cells [120].

## 6 Safety studies

Before introducing clinical trials, safety issues must be carefully controlled for any new promising pharmaceutical agents. To date, only few toxicity studies have been performed with ampelopsin, showing that this natural compound did not induce any acute toxicity or significant side effects in mice at doses ranging from 150 mg/kg to 1.5 g/kg body weight [121]. Although this work clearly indicates that ampelopsin possesses very low toxicity, more studies are needed to establish safety limits and determine the values of no-observed-adverse-effect level (NOAEL) for this attractive compound. Furthermore, a very recent study confirmed the safety of ampelopsin-containing cationic nanocapsules for topical application; however, these tests were performed with cell lines and not in animal models [122]. Therefore, further studies on the safety of ampelopsin are definitely needed.

## 7 Conclusion

In this review article, diverse anticancer effects of the flavanonol ampelopsin are described, focusing on its anti-inflammatory, antiproliferative, proapoptotic, antiangiogenic and antimetastatic activities. The knowledge presented in this paper may be important for further unraveling the molecular pathways regulated by this biomolecule, but could also help to modify the structure of ampelopsin for enhancing its activities. In the future, further toxicity studies should be performed to ensure the safe application of this attractive phytochemical. Nanotechnology based methods could also be opted to decrease required doses of ampelopsin as well to enhance targeted delivery in cancer microenvironment.

## Declaration of Competing Interest

The authors declare that they have no known competing financial interests or personal relationships that could have appeared to influence the work reported in this paper.
